# The Validity and Reliability of the Chula COVID-19 Psychosocial Home Isolation Evaluation Tool (CCPHIET)

**DOI:** 10.7759/cureus.25224

**Published:** 2022-05-22

**Authors:** Paul Thisayakorn, Napat Sirinimnualkul, Yanin Thipakorn, Jirada Prasartpornsirichoke, Chumpol Suraphaphairoj, Gompol Suwanpimolkul, Tippamas Taechawiwat, Jose R Maldonado

**Affiliations:** 1 Department of Psychiatry, Faculty of Medicine, Chulalongkorn University, Bangkok, THA; 2 Department of Psychiatry, King Chulalongkorn Memorial Hospital, Thai Red Cross Society, Bangkok, THA; 3 Department of Medicine, Faculty of Medicine, Chulalongkorn University, Bangkok, THA; 4 Department of Psychiatry, Chulabhorn Hospital, Bangkok, THA; 5 Psychiatry and Behavioral Sciences/Psychosomatic Medicine, Stanford University School of Medicine, California, USA

**Keywords:** psychosocial assessment, reliability and validity, self-quarantine, patient isolation, covid-19

## Abstract

Background: The growing number of cases presenting with COVID-19 during the pandemic has led to a significant shortage of hospital beds. Many patients may not require hospitalization and can be clinically observed in home settings. We have identified a set of psychosocial factors that correlate with unsuccessful home isolation (HI), which in turn might negatively affect the transmission control in the community. Therefore, we developed the Chula COVID-19 Psychosocial Home Isolation Evaluation Tool (CCPHIET), a new screening tool for assessing the psychosocial suitability for HI. This study examines the CCPHIET’s validity and reliability.

Methods: This cross-sectional descriptive study included COVID-19 patients who were deemed to be medically safe for 14-days of HI. The CCPHIET is comprised of eight clinical domains pertinent to HI behavioral compliance and risk for non-adherence. We explored its statistical validity and reliability and discussed the potential utility of this tool.

Results: A total of 65 COVID-19 patients participated in this study. Most patients (58.5%) were deemed to be appropriate candidates for HI according to the CCPHIET. The results of this study demonstrate that the CCPHIET has an acceptable content validity (IOC index > 0.5), moderate internal consistency (Cronbach’s alpha = 0.611) and substantial to excellent inter-rater reliability (Intraclass correlation coefficient = 0.944, Cohen’s kappa= 0.627).

Conclusions: CCPHIET is an easy-to-use tool for assessing the psychosocial suitability of patients advised for at-home isolation with mild and asymptomatic COVID-19. Its implementation can assist clinicians in identifying and redirecting resources to patients at the highest risk for breaking quarantine and save on unnecessary, costly absolute institutional quarantine for those deemed to be psychosocially fit for full adherence.

## Introduction

The third wave of the COVID-19 pandemic in Thailand started in April 2021. The cumulative number of cases had increased 10 times from 29,000 cases in early April to 290,000 cases in early July 2021 [1]. This sharp rise in cases raised concerns about the shortage of medical resources throughout the country. There had been continuous reports of hospital bed shortages in both intensive care and general medicine units. Some hospitals were forced to limit the rate of COVID-19 testing, given initial regulations mandating medical institutions to take responsibility for patients who tested positive at their respective facilities [2]. In turn, limitations on COVID-19 testing further hampered the effectiveness of pandemic control [3]. Rising COVID-19 cases and the lack of COVID-19 beds also lead to increased burnout rates, as well as other physical and psychological negative impacts on the wellbeing of health care personnel [4], including an increase in the number of suicides [5]. COVID-19 also resulted in living in isolation and quarantine leading to an increase in psychiatric symptoms and complications [6].

It is estimated that the proportion of pre-symptomatic/asymptomatic COVID-19 patients ranges between 15.6-45% [7,8]. When mild symptomatic cases are included in the total count, about 80-90% of all COVID-19 cases are uncomplicated [9]. Severe disease, on the other hand, is reported in approximately 10-20% of all cases [10]. Therefore, a significant majority of COVID-19 cases do not require inpatient hospitalization and can be cared for in at-home settings.

There has been a debate about what is the best infectious control strategy for the management of asymptomatic/mildly symptomatic COVID-19 patients. Reports from China demonstrate that containing most of the infected cases in hospitals or dedicated health isolation facilities would greatly reduce household and community transmission in later weeks [11]. However, these strict institutional isolation policies consume an enormous amount of resources and require strong law enforcement. In addition, institutional isolation may stir up problematic psychological issues when individuals were held in unfamiliar hospital environments for 14 days [12]. In contrast, the United States of America and European countries applied home isolation (HI) measures for asymptomatic/mildly symptomatic COVID-19 cases, which allow them to spare hospital beds and medical resources that could be used for the management of severe patients [13,14]. India implemented similar HI policies which resulted in an 80% reduction in COVID-19 hospitalized bed requirements [15]. Yet clinical experience, supported by available data, indicates that a sizable number of COVID-19 patients might not be able to follow self-isolation quarantines, thus increasing the risk of spreading the virus to others. Reports from the United Kingdom and Israel demonstrated a high home quarantine failure rate of up to 43% and 75%, respectively [16,17]. Applying the infectious simulation model, Dickens et al. concluded that institutional-based isolation is more efficacious than home-based isolation in terms of transmission control [18]. Despite these findings, it seems impractical to implement absolute institutional isolation in Thailand given the steeply rising number of new cases since June 2021, which have overloaded our hospitals’ capacity between July and September 2021. Thus, we propose to create a model or tool that could provide a middle ground between home and institutional isolation practices, while still minimizing community transmission control and alleviating medical resource shortages. 

In the field of Transplantation Psychiatry, all solid organ transplant candidates are required to undergo psychosocial evaluation by transplant psychiatrists or psychologists to assess the overall psychological, behavioral, and social fitness and risks [19]. The standard psychosocial assessment tools commonly used in the field of Transplant Psychiatry are the Psychosocial Assessment of Candidates for Transplantation (PACT, 1989) [20], the Transplant Evaluation Rating Scale (TERS,1993) [21], and the Stanford Integrated Psychosocial Assessment for Transplantation (SIPAT, 2012) [22,23]. The main purpose is to systematically assess the patient’s knowledge, capacity for adherence, associated health behaviors, availability and functionality of their support system, the appropriateness of their home environment, presence of psychopathology, coping styles, and any past or present substance use problems. This assessment allows transplant teams to identify and categorize each candidate’s unique psychosocial risks and readiness level, which allows for both risk stratification and the development of a customized intervention plan to meet the candidate’s personalized needs.

Here we propose the development of an instrument that would similarly provide an assessment of the psychosocial fitness of any given individual with asymptomatic/mildly symptomatic COVID-19 patients to safely and successfully adhere to the 14-day HI protocol. Conversely, such an instrument would allow clinicians to predict those patients likely to fail home isolation protocols and plan for alternate, in-hospital isolation. Ideally, such a tool could also assist in the identification of potential psychosocial and environmental risks that should be addressed by the clinical team before patients can be safely discharged to their home environments.

To address this need, our research team developed a new psychosocial assessment tool, using as the basis the concepts already developed by existing pre-transplant psychosocial evaluation tools, customized by the knowledge of the positive and negative factors that correlate with success to HI protocol adherence. The resulting tool is the “Chula COVID-19 Psychosocial Home Isolation Evaluation Tool” (CCPHIET). If our original findings are successfully replicated, we expect the CCPHIET to be part of the solution regarding the dilemma between strict and costly absolute institutional quarantine versus the potentially unsuccessful absolute HI. We intend to take advantage of the current COVID-19 crisis in Thailand, to study the validity and reliability of the CCPHIET among asymptomatic or mildly symptomatic Thai COVID-19 patients whose clinicians have recommended HI as part of their treatment plan.

## Materials and methods

Study design, setting, and population

We designed a cross-sectional descriptive study to develop an original tool for the psychosocial assessment of the suitability of patients diagnosed with COVID-19 to undergo and succeed in adhering to a strict HI protocol. Subsequently, we intend to examine the new instrument’s validity and reliability.

The study population consisted of patients who had a positive Nucleic Acid Amplification Test (NAAT) for SARS-CoV-2 infection by using Reverse Transcriptase Polymerase Chain Reaction (RT-PCR) according to the WHO case definition [24]. Subjects’ information was collected between August and September 2021 using the King Chulalongkorn Memorial Hospital electronic medical records (EMR) system, in Bangkok, Thailand. Asymptomatic or mildly symptomatic COVID-19 patients were assessed by internal medicine physicians to be physically safe and appropriate for the 14-day COVID-19 HI protocol. Two major subgroups enrolled in this study: patients who had recently tested positive for COVID-19 and were instructed to receive home isolation (post-swab HI), and patients who had been admitted to the hospital for treatment, but subsequently deemed medically stable for discharge before completing 14 days of institutional quarantine (post-discharge HI). Both groups of patients were required to self-isolate within their residence for an additional 14 days. Only patients between 18-60 years of age and meeting the above-described criteria were included. The exclusion criteria included: patients who developed moderate to severe symptoms of COVID-19 infection during HI, those unable to cooperate with the psychiatric interview (e.g., dementia, delirium, intellectual disability, patients experiencing active, severe psychiatric conditions posing risk to self or others upon discharge, those unable to communicate in the Thai language), and subjects unwilling to participate in the study. Patients unable to use smartphones and online chat programs were also excluded because video calls were necessary for conducting the interview. 

Participant recruitment

Patients whose physical condition did not require hospitalization, as judged by their internists, were contacted by the investigators via video call and informed about the study protocol along with potential benefits and risks. Also, patients who were initially admitted but whose physical conditions have improved sufficiently for home care before completing institutional quarantine were contacted in the same manner. Patients willing to participate in the study were interviewed according to the CCPHIET questionnaire by the study investigator. According to the Institutional Review Board of our faculty, patients could give consent by action and no physical signature was required. 

Development of the CCPHIET

Researchers reviewed the literature regarding COVID-19 symptom characteristics, transmission methods, natural illness course, available prevention protocols, and HI policies [25-29]. Based on currently recommended clinical domains by the Center for Disease Control and Prevention, as well as the recommendations of Thailand's Ministry of Public Health [30-31] and based on the successful experience of solid organ transplant psychosocial evaluation tools, our research team chose eight clinical domains that were likely to be relevant predictors of successful adherence to HI protocols. For example, current self-quarantine literature, government HI protocol, and transplant evaluation tools all identify knowledge about the disease as an important factor in evaluating an individual’s health care adherence. Therefore, this factor was included in one of the eight clinical domains of the CCPHIET.

The eight clinical domains were (1) appropriateness of the housing and surrounding environment, (2) the subject’s knowledge of COVID-19 symptoms and illness pattern, (3) the patient’s ability to provide basic medical care for self at home, (4) the patient’s commitment and cooperation in community transmission prevention, (5) a history of prior positive health care behaviors, (6) adequate social support system during the home quarantine, (7) presence of psychopathology, and (8) past and current history of substance use problems.

The research team proceeded to create potential questions to assess these domains based on the existing COVID-19 and transplant literature. Subsequently, we reviewed all questions and items of each domain and shaped them to be objective, specific, and relevant to HI compliance and risk for adherence failure. The tool instructions, contraindication items, and scoring system were also developed using the same process.

Each of the eight major clinical domains contained five specific questions, each question having equal weight, that is one point. Points were accumulated when patients described a correct, suitable, or appropriate answer for each question. The possible maximum CCPHIET score is 40. A tentative score grading was estimated by the researchers, with a score of 33-40 indicating a “good candidate” for home quarantine and expected to be highly compliant with the home isolation without creating further community disease transmission. Patients who scored between 25-32 were deemed to be “minimally acceptable”, suggesting that some psychosocial risks were identified and should be addressed in order to improve suitability for HI. Those scoring less than 25 were considered to be high-risk candidates, in which case the medical team should consider institutional care due to identified psychosocial factors that might interfere with full HI adherence. The CCPHIET assessment was designed for administration via online video or telephone interview and to be conducted by medical professionals with some background in mental health or social work. Yet, we predict that with minimal training requirements general health workers could also use this tool if there is an urgent need in each community.

Questionnaire validation

The research validation team consisted of two experienced staff psychiatrists, an infectious disease staff physician, a transplant nurse coordinator, and a senior social worker (five examiners in total). They all examined and reviewed each item of the CCPHIET for content validity. Item-object congruence (IOC) was rated by each content expert, and questions with IOC less than 0.5 were adjusted accordingly.

Investigators interviewed all patients using the CCPHIET questionnaire via a video chat application. Each patient encounter was scored by two raters. The video interview lasted between 8-15 minutes per patient. Questionnaire items were asked as open-ended questions, with further clarification if necessary. Raters then interpreted the patients’ answers and scored each item in a yes/no format. Demographic data, including age, gender, marital status, years of education, and monthly income was also collected at the time of the interview. Results from the CCPHIET video psychosocial evaluation and recommendations were then shared with the infectious disease specialists and medical HI coordinators, so they could make a final decision regarding the HI treatment. Any identified psychosocial risk factors were cooperatively managed by the medical HI clinicians, social work personnel, and the research team psychiatrists. Both post-swab/post-discharge HI patients were still in contact with their HI clinicians/coordinators throughout the isolation period in case any urgent medical needs were to arise. 

Statistical analysis

Demographic data were summarized as frequency, mean, or median, as appropriate. Inter-rater reliability was calculated with intraclass correlation using a two-way random-effects model and Cohen’s kappa. Cronbach’s alpha was used to measure internal consistency. Spearman’s rank correlation coefficient was used to explore the correlation between demographic data and CCPHIET scores. All data were analyzed with SPSS version 22.0 (IBM Corp., Armonk, NY).

Ethical approval

Approval was obtained from the Institutional Review Board of the Faculty of Medicine, Chulalongkorn University, Bangkok, Thailand on 18th August 2021 (Registration number 620/64, Certificate of approval number 1168/2021), in compliance with the International Guideline for Human Research protection, as required by the Declaration of Helsinki, was conducted according to the Thai and International ethics and privacy laws. This article was previously posted to Preprints.org on October 20, 2021.

## Results

Patient characteristics

A total of 125 patients were approached for recruitment into this study. The most common reason for exclusion was the patient’s unwillingness to participate (37 patients). Eleven patients did not know how to join a video call. Technical errors disrupted the completion of the interview with 12 participants. The final sample population consisted of 65 patients. Most patients (>60%) were female, with ages ranging from 18 to 60. More than half of our study population had no more than 12 years of formal education. The median monthly income was 13,000 Thai Baht, which is considered a low-to-middle socioeconomic class. There were nine participants without current income, including two participants who were students, and seven participants who were currently unemployed (Table 1).

**Table 1:** General characteristics of study participants SD: standard deviation; IQR: interquartile range; *Income in Thai Baht

Characteristic	
Gender, number (%)	
Male	25 (38.5)
Female	40 (61.5)
Age, mean ± SD	35.1 ± 10.8
Marital status, number (%)	
Single	30 (46.2)
Married	35 (53.8)
Education, number (%)	
0-6 years	14 (21.5)
7-12 years	23 (35.4)
Vocational education	7 (10.8)
Bachelor’s degree or above	21 (32.3)
Monthly income*, median (IQR)	13000 (10000, 20000)
Employment (%)	
Employed, with income	56 (86.2)
No current income	9 (13.8)

Questionnaire characteristics

**Figure 1:**
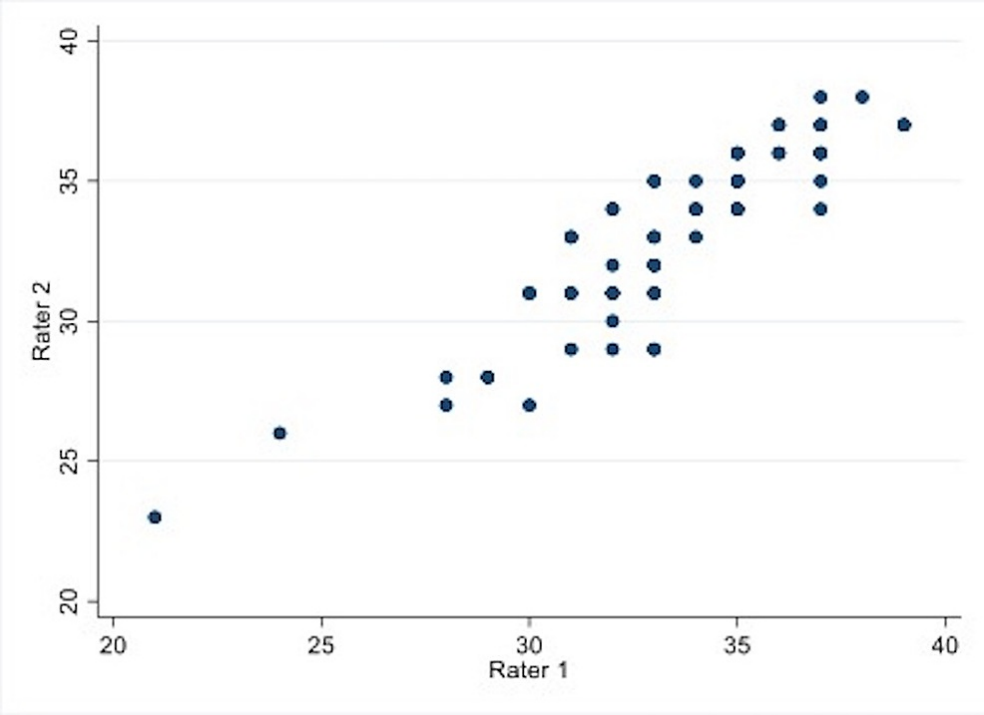
Scatter plot of agreement between two independent raters

The item-object congruence of all items in the final questionnaire was greater than 0.5. The Cronbach’s alpha demonstrated an internal consistency of 0.611 (95%CI 0.462, 0.735), which is within the acceptable range [32]. For interrater reliability, the total score of the 65 patients rated by two independent raters (psychiatrists) was analyzed using the intraclass correlation coefficient (ICC). The two-way random-effect model on absolute agreement ICC of the two raters was 0.944 (95%CI 0.907, 0.966) which represents an excellent level of reliability [33]. High consistency was observed in the scatter plot graph (Figure 1). Inter-rater agreement on categorical measurement (good candidate, minimally acceptable candidate, and at-risk candidate) was substantial using Cohen’s Kappa statistic (0.627, 95%CI 0.435, 0.820) [34].

Suitability for home isolation

**Figure 2:**
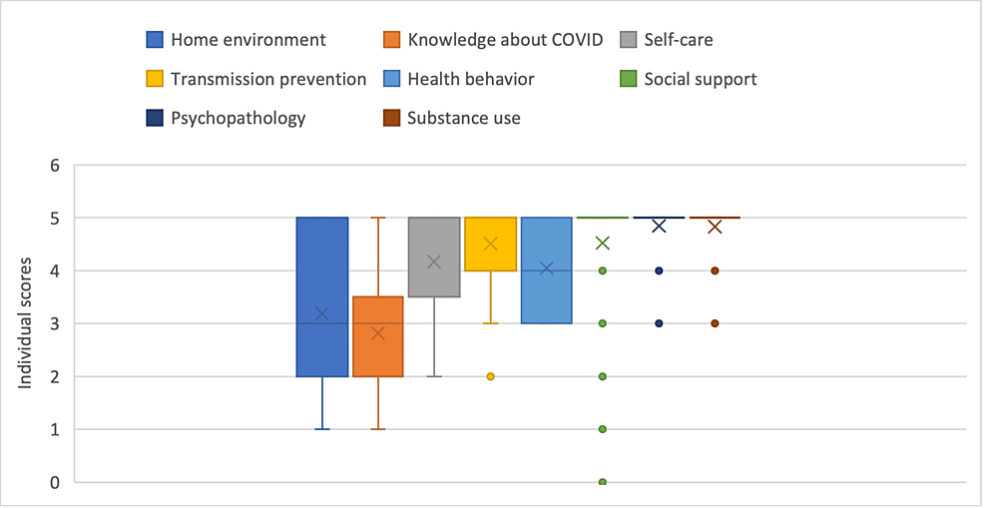
Box-and-whisker plot of scores in each domain As displayed in order from left to right: (1) home environment, (2) knowledge about COVID-19 symptoms, (3) self-care, (4) commitment to transmission prevention, (5) health behavior, (6) social support, (7) psychopathology, and (8) substance use

A significant portion of evaluated patients (58.5%) were deemed as good candidates for HI based on the CCPHIET assessment. Only one patient was evaluated as ‘at risk’ for non-adherence to HI, obtaining a total score of 23. The median total score was 34 (IQR 31, 35) out of a potential maximum score of 40. The majority of patients demonstrated no psychopathology, good social support, and no evidence of substance abuse, which might interfere with adhering to full HI, based on most patients (80-88%) receiving a full score of five in each of these domains. Potential commitment to transmission prevention was also considered to be good (mean score 4.5). Our study population had moderate knowledge about COVID-19 symptoms (mean score 2.8) while demonstrating better knowledge about self-care (mean score 4.2). Health maintenance behaviors, including the regular use of masks, maintaining social distancing, and general health maintenance habits, were generally good (mean score of 4.0). The suitability of home environment was more diversified, with almost half of the sample (47.7%) receiving a score of 4-5, while 40% of patients scored 1-2 (Figure 2).

Higher education levels correlated with better health behaviors (Spearman’s rho 0.294, p = 0.017), while higher income correlated with better home environments (Spearman’s rho 0.262, p = 0.035) and more extensive knowledge about COVID-19 (Spearman’s rho 0.298, p = 0.016). It is likely that these factors led to the positive correlation seen between education and income with higher total scores observed on their CCPHIET assessment. Of note, in our sample, being married was correlated with lower scores on health behavior (Spearman’s rho -0.314, p = 0.011), which may be due to more difficulty in social distancing and fewer opportunities for general health maintenance.

## Discussion

We developed a new screening tool (the CCPHIET) with the aim to systematically assess the psychosocial suitability for HI and correctly identify asymptomatic/mild symptomatic COVID-19 patients with a high potential for HI success. The CCPHIET has an acceptable content validity (IOC index > 0.5), moderate internal consistency (Cronbach’s alpha = 0.611), and substantial to excellent reliability (ICC 0.944, and Cohen’s kappa= 0.627).

In our sample, most study subjects were married, employed, and middle-aged females with low to moderate education and income level. These demographic characteristics are representative of the overcrowded communities in and around Bangkok, one of which was most affected by the delta variant during the study month. Being married, having lower education and poor income were negatively correlated with higher health behavior scores and CCPHEIT total scores, therefore. Almost 60% and 40% of the COVID-19 patients in this study were categorized as appropriate, and minimally acceptable candidates for HI by the CCPHIET scoring system.

We propose that by teaching important health care information and arranging for adequate home support, medical personnel might be able to improve many of the risk factors that contribute to low CCPHIET observed among minimally acceptable candidates. If this hypothesis is correct, these interventions might assist in making this population more suitable candidates for the HI protocol, thus potentially significantly increasing (>60%) the percentage of COVID-19 asymptomatic/mild symptomatic patients that could be managed in the HI settings. The median total CCPHIET score of 34/40 from the study cohort was in the appropriate candidate range, as the purpose of this tool is to detect patients who can provide good self-care and have a high level of compliance with COVID-19 prevention at home.

Less than 10% of the patients in our cohort reported psychopathology and substance use. This number was significantly lower than the 20-30% proportion of COVID-19 Thai patients in field hospitals who have been reported to suffer from depression and anxiety [35] or the 25% proportion of mental health and substance use comorbidity found in the hotel-based isolation cohort in a sample of homeless patients in San Francisco, USA [36]. Up to 88% of our sample was found to have a good support system, which assured that they would have adequate physical and psychological support from their family, friends, and extended community. The availability of a support system was identified as an important factor for HI success in previous studies [17,29,37]. Patients in our study also demonstrated moderate to good level knowledge of COVID-19 symptoms, self-care, and prevention methods. These factors have been associated with good quarantine adherence, as described in previous studies [38-40]. Nearly half (40%) of the patients scored less than 3 points in the home environment domain. Many COVID-19 home care resources recommend that the setting for HI should include separate bedrooms and bathroom facilities for quarantined patients, or at least have enough space to accommodate social distancing requirements and the ability to disinfect the shared area [41].

There are some limitations to our study. The recruited patient numbers are small, which might affect the overall reliability of the CCPHIET. Moreover, 62 out of 125 patients were initially excluded from the study participation mostly due to unwillingness to be involved in the study; they reported they were feeling too unwell, too occupied with household adjustments, or uncomfortable with technology use. This may be viewed as an exclusion bias of this study. Our sample did not include patients likely to have adverse psychosocial and environmental backgrounds as these patients would be admitted into hotel quarantine rather than home isolation, which was not under the direct care of our system at that time. All of the home isolated patients were followed by the medical home health care team until day 14. Unfortunately, we did not collect behavioral outcomes at the end of the isolation period. These limitations were due to our hospital regulations, the scarcity of research manpower, and the time constraints during the pandemic while aiming to rapidly develop this tool. We only demonstrated the content validity of the CCPHIET but did not report other validity values such as the sensitivity and specificity, given the lack of other accepted external standards of suitability for home isolation. The Cronbach’s alpha was in the low range of an acceptable value, but this might be due to the multidimensional nature of our questionnaire. The low number of subjects may have also negatively affected Cronbach’s alpha. The original CCPHIET is in the Thai version and was examined in Thai patients. The questionnaire has been translated into English, although the English version has not yet been validated. Future directions for the CCPHIET development would be to fully implement it into the real world COVID-19 medical system and explore its predictability and utility, impact on transmission control, and as well as economic benefits on a larger scale.

**Figure 3:**
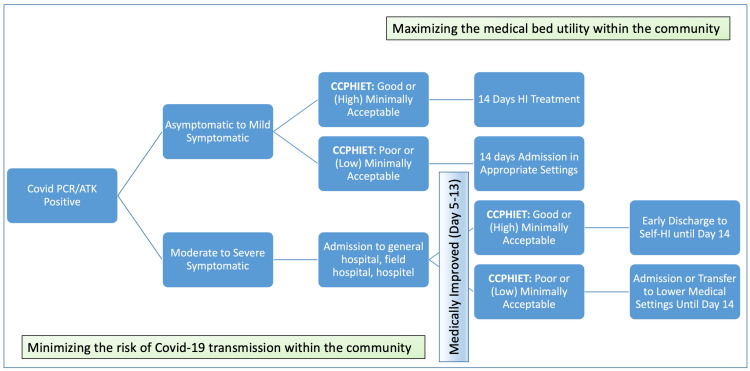
Proposed diagram of CCPHIET use in the care of patients with COVID-19 PCR: polymerase chain reaction for COVID-19; ATK: antigen test kit for COVID-19; CCPHIET: The Chula COVID-19 Psychosocial Home Isolation Evaluation Tool

Along with the development and validation of this tool, we learned that the CCPHIET helped categorize the post-swab COVID-19 patients into psychosocially appropriate, minimally acceptable, poor, or contraindicated candidates for HI. This psychosocial information would assist physicians in matching each patient to the most suitable medical setting, whether it is institutional treatment or home-based treatment. Moreover, this tool could also help physicians consider the possibility of early home discharge for symptomatic COVID-19 patients who are symptomatically improved, without the need to contain them within the medical unit for the full duration of 14 days, while still minimizing community transmission (Figure 3). The risks identified by CCPHIET screening should assist physicians in educating patients about factors associated with poor outcomes, while addressing or adjusting present risk factors, in order to reduce the possibility of community transmission after hospital discharge.

## Conclusions

The Chula COVID-19 Psychosocial Home Isolation Evaluation Tool (CCPHIET) is a newly developed psychosocial assessment tool designed to identify asymptomatic/mild symptomatic COVID-19 patients who would be appropriate candidates for HI and select symptomatic COVID-19 patients who were improving for early home discharge. Implementing this tool into the COVID-19 care system might increase the possibility of safer and more successful HI outcomes for both post-swab and post-discharge COVID-19 patients.

## Appendices

Chula Covid-19 Psychosocial Home Isolation Evaluation Tool (CCPHIET)

Date: ......................................................

Name ................................................  Gender ...................  Age ................... Marital Status ....................................  Occupation ........................................

Education Level .......................  Average Income per Month ..........................

Instructions: This assessment tool is for use by psychiatrists, psychologists, psychiatric nurses, social workers, mental healthcare professionals, and other healthcare professionals to acquire information on the psychosocial aspects of COVID-19 patients to evaluate the appropriateness and safety of home isolation, without transmitting the virus to acquaintances and communities. The assessment tool is divided into two sections as follows. 

Section 1: This section evaluates the appropriateness and risks of home isolation by using online interviews, which consists of 8 clinical domains; 40 questions in total. If patients can answer the key words, or the answer is satisfactory according to the criteria of each question, the assessor will put a check in the box, sum up the scores, then interpret in the section 2.

Section 2: This section considers absolute contraindications of the psychosocial aspect of home isolation, scoring criteria, risk identification, and suggestions.

This assessment tool; however, is the primary evaluation of the psychosocial aspect to acquire information to be combined with their physical condition and other aspects of each patient so the primary team may identify the psychosocial risk factors of each patient, and be able to choose the most appropriate and safest setting to deliver treatment to the COVID-19 patient with utmost competence of each medical system.

**Table 2:** The Chula COVID-19 Psychosocial Home Isolation Evaluation Tool (CCPHIET) questionnaire

Section 1: The psychosocial aspects which are taken into consideration when evaluating the appropriateness of home isolation of COVID-19 patients	Total	
		1) Appropriateness of accommodation and surroundings (Each item is 1 point; 5 points in total)		
What is your accommodation like?			
​​​​​​​​​​ 1.1 The patient or family owns or rents accommodation. (1 point)			
1.2 The accommodation is not located in a slum. (1 point)			
1.3 The patient does not need to share communal areas with other family members. (1 point)			
Do you have your own private bedroom and bathroom?			
1.4 The patient has his/her own private bathroom. (1 point)			
1.5 The patient has his/her own private bedroom. (1 point)			
2) Cooperation in preventing the transmission of COVID-19 to others (Each item is 1 point; 5 points in total)		
Do you need to leave your accommodation or is there an occasion you have to contact others during home isolation?		
2.1 The patient does not need to leave his/her home isolation area to run errands, buy food and necessities, or take care of people outside the home isolation areas. (1 point)		
2.2 The patient does not need to physically contact others such as children or the elderly during home isolation. (1 point)		
What are your plans on eating, cleaning utensils, washing clothes, and disposing of waste?		
2.3 The patient will have someone provide food and can eat alone. (1 point)		
2.4 The patient can clean utensils, wash clothes, and clean room, or can have someone do it for him/her safely. (1 point)		
2.5 The patient will separate, tightly tie trash bags, and safely dispose of waste. (1 point)		
3) Capacity of social support system during home isolation (Each item is 1 point; 5 points in total)		
How many people can assist you are there in the accommodation? Who are they?		
3.1 The patient has at least 1-2 assistants. (1 point)		
3.2 The patient has close supporters (family members, close friends, or close colleagues). (1 point)		
Are the supporters able and ready to take care of you?		
3.3 The supporter(s) have received advice and should understand how to take care of a COVID-19 patient at home. (1 point)		
3.4 The supporter(s) should be responsible, and able to provide necessities for the patient and not have serious medical conditions. (1 point)		
3.5 The supporter(s) can get to the patient within 1-2 hours if help is needed. (1 point)		
4) Knowledge and understanding of COVID-19 (Each item is 1 point; 5 points in total)		
What are the symptoms of COVID-19 infection?		
4.1 fever, cough, or sore throat (1 point)		
4.2 loss of smell or taste (1 point)		
4.3 headache, muscle soreness, or chest tightness (1 point)		
4.4 shortness of breath, or difficulty breathing (1 point)		
4.5 diarrhea, or lethargy (1 point)		
5) Self-care capacity during home isolation (Each item is 1 point; 5 points in total)		
How will you monitor your symptoms each day?		
5.1 The patient will monitor for fever, or will use a thermometer. (1 point)		
5.2 The patient will monitor for shortness of breath, or will use a pulse oximeter. (1 point)		
If you have mild symptoms of COVID-19, how will you take care of yourself?		
5.3 The patient will take antipyretics / mucolytics / rest and sleep. (1 point)		
If you have severe symptoms of COVID-19, how will you contact and go to a hospital? What hospital?		
5.4 The patient can name their primary hospital. (1 point)		
5.5 The patient can contact an ambulance, or go to the hospital without using public transportation.		
6) Health behaviors before getting infected with COVID-19 (Each item is 1 point; 5 points in total)		
During the past month, what were your health behaviors before getting infected with COVID-19?		
6.1 The patient always wore masks. (1 point)		
6.2 The patient usually washed his/her hands. (1 point)		
6.3 The patient always practiced social distancing. (1 point)		
Do you have any medical problems? If so, are they properly treated?		
6.4 The patient does not have any medical conditions, or regularly takes medicines, gets treatment, and goes for follow up on his/her medical problems. (1 point)		
In general, how do you take care of yourself?		
6.5 The patient exercises, eats healthy food, and gets enough sleep. (1 point)		
7) Alcohol and drug abuse (Each item is 1 point; 5 points in total)		
Do you drink alcohol or smoke regularly? What are the amounts and frequency of alcohol intake or nicotine use? Do you have history of alcohol withdrawal?		
7.1 The patient does not drink alcohol to the point that it disturbs work or negatively affects his/her current relationship with others. (1 point)		
7.2 There is no history of alcohol use disorder. (1 point)		
7.3 There is no history nicotine use disorder. (1 point)		
*Have you had history of illicit drug addiction or have you used drugs that contain addictive substances? (amphetamines, marijuana, kratom, opioids, cough medicines, sleeping pills, etc.) *		
7.4 The patient does not have a substance use disorder nor has undergone drug rehabilitation. (1 point)		
7.5 The patient is not addicted to narcotics or drugs that contain addictive substances. (1 point)		
8) Psychopathology (Each item is 1 point; 5 points in total)		
During the past two weeks, did you feel totally sad, depressed, hopeless, or have little interest in doing things?		
8.1 The patient does not have major depressive symptoms. (1 point)		
During the past two weeks, did you feel very nervous, anxious, or could not stop worrying?		
8.2 The patient does not have anxiety disorder symptoms. (1 point)		
Are you receiving a psychiatric treatment?		
8.3 The patient is not receiving any psychiatric treatments, or is receiving treatment with stable symptoms. (1 point)		
During the past two weeks, did you have suicidal thoughts or want to harm yourself?		
8.4 There is no report on suicidal thoughts or self-harm. (1 point)		
Does the patient hide or distort unfavorable information?		
8.5 There is no evidence from the medical record or during the interview that indicates intentional distortion of unfavorable information . (1 point)		
Total Score (40 points)		

Version 2.2 Dec 2021

Developed by P. Thisayakorn, N. Sirinimnualkul, Y. Thipakorn, et al. Received research grant funding from Faculty of medicine, Chulalongkorn University, Thailand. No permission required to translate, display, or distribute.
